# Opto-SICM framework combines optogenetics with scanning ion conductance microscopy for probing cell-to-cell contacts

**DOI:** 10.1038/s42003-023-05509-3

**Published:** 2023-11-08

**Authors:** Qianqian Song, Anita Alvarez-Laviada, Sarah E. Schrup, Benedict Reilly-O’Donnell, Emilia Entcheva, Julia Gorelik

**Affiliations:** 1 Imperial College London, Du Cane road, W12 0NN London, UK; 2 Department of Biomedical Engineering, George Washington University, Washington, DC USA

**Keywords:** Optogenetics, Heart failure

## Abstract

We present a novel framework, Opto-SICM, for studies of cellular interactions in live cells with high spatiotemporal resolution. The approach combines scanning ion conductance microscopy, SICM, and cell-type-specific optogenetic interrogation. Light-excitable cardiac fibroblasts (FB) and myofibroblasts (myoFB) were plated together with non-modified cardiomyocytes (CM) and then paced with periodic illumination. Opto-SICM reveals the extent of FB/myoFB-CM cell-cell contacts and the dynamic changes over time not visible by optical microscopy. FB-CM pairs have lower gap junctional expression of connexin-43 and higher contact dynamism compared to myoFB-CM pairs. The responsiveness of CM to pacing via FB/myoFB depends on the dynamics of the contact but not on the area. The non-responding pairs have higher net cell-cell movement at the contact. These findings are relevant to cardiac disease states, where adverse remodeling leads to abnormal electrical excitation of CM. The Opto-SICM framework can be deployed to offer new insights on cellular and subcellular interactions in various cell types, in real-time.

## Abstract (web summary)

Opto-SCIM combines scanning ion conductance microscopy with optogenetic interrogation to study the hetero-cellular interactions between cardiac fibroblasts or myofibroblasts and cardiomyocytes.

## Introduction

High-resolution live cell imaging and manipulation within multicellular context in a contactless and non-interfering manner is the holy grail of understanding biological processes. Optical methods have developed very rapidly to provide ever improving space-time resolution, often beating the diffraction limit. However, to date very few imaging modalities have the potential to map nanoscale details in live, moving cells–details on par with the information provided by electron microscopy on fixed cells. One such technique, proposed by Hansma in 1989 is the Scanning Ion Conductance Microscopy, SICM^1^. This contactless method measures the ion current at the tip of an electrolyte-filled nanopipette as the pipette approaches the cell surface. Within the proximity of the cell, ion flow decreases due to steric constraints and therefore, the ion current reflects z-position. With a real-time feedback control to maintain a constant distance from the surface while scanning, a nanoscale topographic image is obtained in SICM. While similar in output to Atomic Force Microscopy (AFM), SICM is more physiologically compatible as it applies much smaller forces compared to AFM^2,3^. The utility of SICM for imaging live cells has been illustrated in series of studies and important technical developments, starting in 1997^4^. It has been combined with confocal microscopy and fluorescence imaging^5^, including Foster Resonance Energy Transfer (FRET-SICM) for location-specific readouts of the responses of secondary messengers by FRET^6^. A key development allowing improved resolution (down to 20 nm) and robustness in imaging sharply varying in height cell surfaces is the hopping-mode SICM^7^ that allows tracking of surface movement at reasonable speed; this version of SICM is used in the current study. New biological insights have been obtained with the technology over the last decade, including a wide range of applications, e.g., hybrid imaging of cell topography and ion channel activity^8–10^, elucidating the cell-surface specific compartmentalization and responses of beta-adrenergic signaling^6,11^, quantifying changes in the intricate T-tubule structures in myocytes and their important remodeling in disease^12,13^, providing highly localized mechanical actuation through the pipette^14^ or using SICM to characterize mechanical properties of live cells^3,15–17^. Furthermore, SICM has been used for delivery of biomolecules^18–20^ and to study the interaction of nanoparticles with cells^21,22^. Specifically, with respect to cardiac utility, SICM can be integrated with several other methods to elucidate the highly dynamic nature of cardiac cells, their physiology and pathologic responses at several spatial scales^23^. The technique is particularly valuable at linking structure and function.

In a parallel development over the last 20 years, a versatile optical manipulation method emerged - optogenetics - which uses genetic expression of light-sensitive ion channels to control biological processes by light with millisecond precision^24–26^. Since its early adoption in cardiac research^27–29^, optogenetics has been applied to a variety of problems related to the function of the heart over the last decade^30^. One of the main advantages of optogenetics compared to other perturbation methods (pharmacological, electrical or mechanical actuation or photo uncaging) is the cell-specific or organelle-specific targeting. It has been demonstrated that optical stimulation can be applied to non-transformed cells of interest through light-sensitized cells as long as the two cell types are electrically coupled–*“a tandem-cell-unit”* operation^29^. This concept has been leveraged to probe the extent of electrical coupling between cardiomyocytes and optogenetically transformed non-myocytes by light. For example, optical triggering of cardiomyocyte responses via sympathetic neurons^31–34^ or parasympathetic neurons^35,36^ revealed the kinetics of autonomic responses and the effects on cardiac wave dynamics. Optogenetics was also used to uncover the importance of macrophage–myocyte electrical coupling in the transmission of signals through the atrioventricular node^37^. Electrical coupling between the most abundant^38^ non-myocyte cell type (fibroblasts) and cardiomyocytes has been a subject of discussion and controversy for a long time^39–41^. It is believed that under stress conditions, such as in the altered mechanical milieu after myocardial infarction, fibroblasts (FBs) undergo a transition to a more contractile phenotype (myofibroblasts, myoFBs)^42^ that is likely to have increased gap junctional expression and increased electrical interactions^41,43,44^ or mechanical interactions^45^ with cardiomyocytes. Strong coupling between these two cell types would have major electrotonic effects on normal cardiac excitation and may present as an arrhythmogenic substrate^46–48^. Considering the importance of these interactions, fibroblast-myocyte coupling has been of great interest, including in optogenetics enabled studies^49–54^.

An integrated optogenetic and SICM system (Opto-SICM), can be a powerful tool in dissecting heterocellular coupling as seen between fibroblasts and cardiomyocytes, under cell-specific optical stimulation and with nanoscale resolution of mapping the contact zone. In a previous study, SICM was used to track the dynamism of fibroblast-myocyte contact under several pharmacological manipulations^55^, with resolution not available using optical microscopy. Here we demonstrate the implementation of Opto-SICM and provide an example application to understand how the extent of physical contact and the dynamics of the contact zone may influence the transmission of light-triggered excitation between fibroblasts or myofibroblasts and cardiomyocytes. Experiments were performed using primary neonatal rat cardiomyocytes and fibroblasts, with optogenetic transformation of the fibroblasts using channelrhodopsin-2 (ChR2). A specific TGF-β receptor I kinase inhibitor, SD208^48,56,57^, was used to prevent the spontaneously emerging myofibroblast phenotype in cell culture on a rigid substrate in order to create fibroblast-cardiomyocyte (FB-CM) and myofibroblast-cardiomyocyte (myoFB-CM) pairs for experiments using Opto-SICM under optogenetic pacing. Using Opto-SICM, we analyze the factors influencing the transmission of excitatory signals in these heterotypical cell pairs.

## Results

### Opto-SICM measurements

**Fig. 1:**
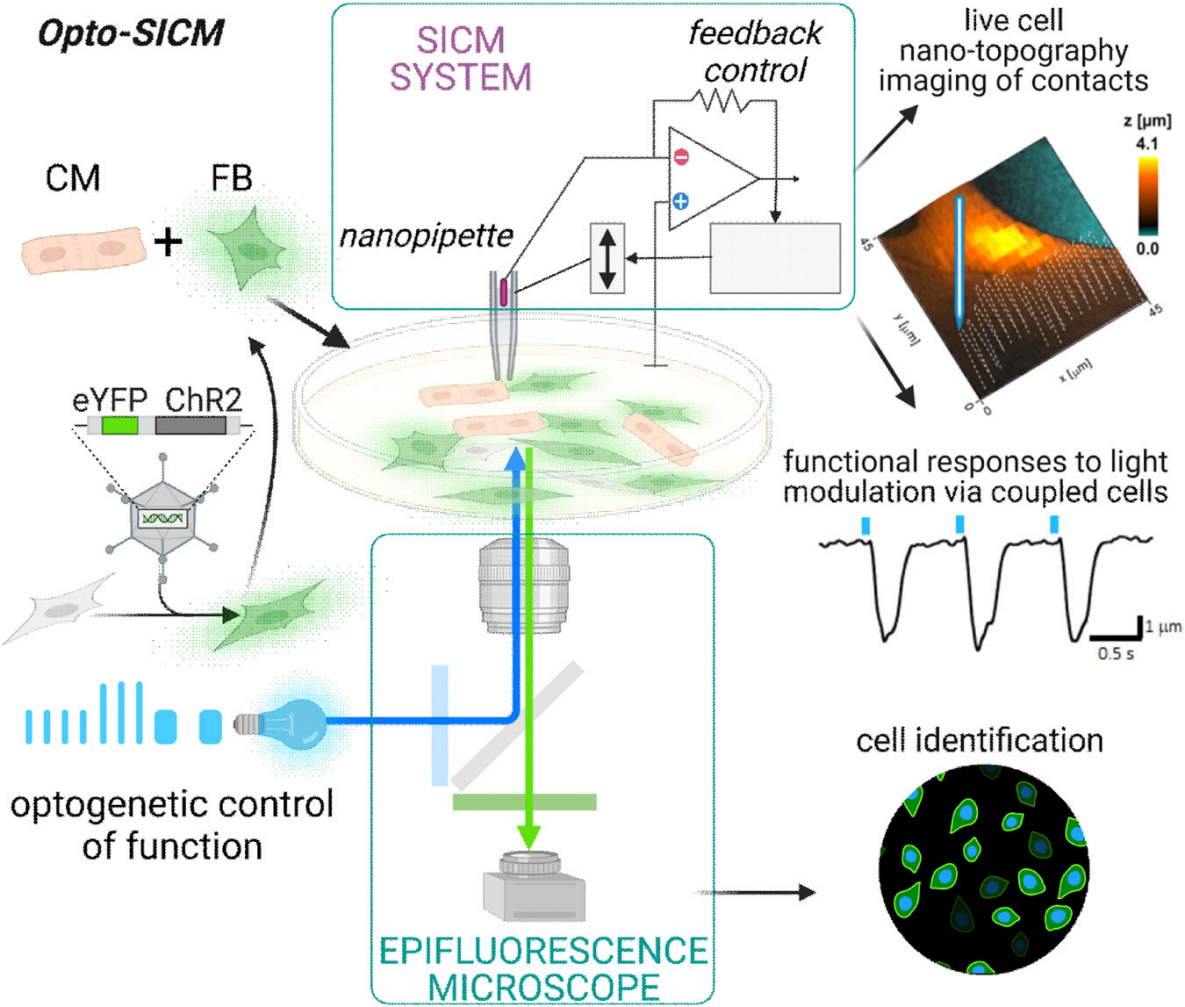
Integrated Opto-SICM system to study heterocellular connections. Cardiac fibroblasts were modified to express ChR2-eYFP by adenoviral vector; the ChR2-FB were sparsely co-cultured with non-transformed CMs. An inverted epifluorescence microscope (bottom) was modified to include a controllable light source for optogenetic stimulation (of ChR2-FBs). The microscope was also used to identify ChR2-FBs in pair with CMs for SICM mapping. The SICM system (top), operating in hoping mode, consists of a nanopipette and a control circuit with a current amplifier and real-time feedback to adjust the z-position of the pipette based on measured current. It outputs nano-topographic images to map the ChR2-FB-CM connections and when recording the vertical displacement of the nanopipette it captures CM contractions in response to the optogenetic stimulation. Biorender was used to create parts of this figure.

**Fig. 2:**
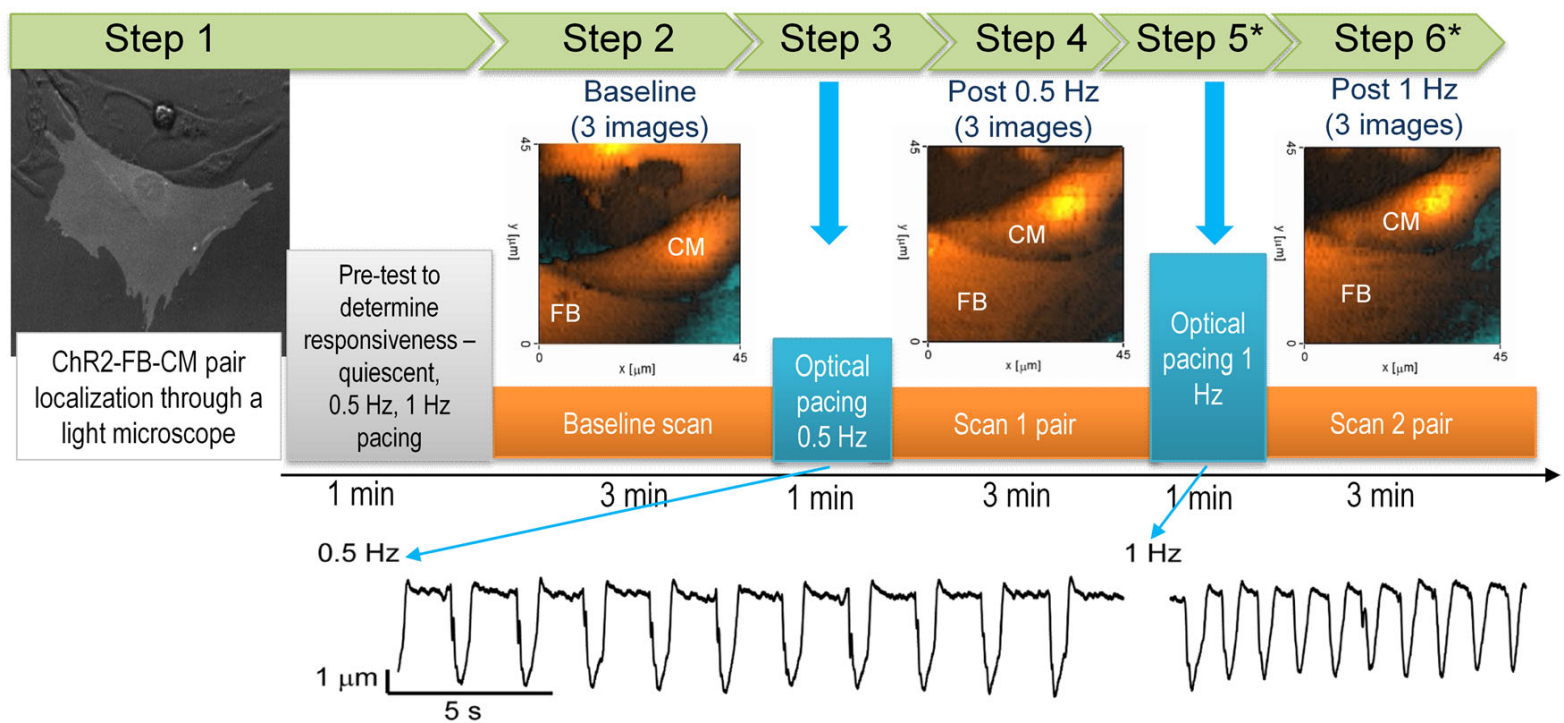
Opto-SICM data acquisition protocol. After localizing a ChR2-FB-CM pair using the epifluorescence microscope (Step 1) and a pre-testing optical stimulation sequence, the pairs were sequentially stimulated at 0.5 Hz (Step 3) and 1 Hz (Step 5). Three sets of topographic images were obtained – baseline scan (Step 2), a scan after 0.5 Hz pacing (Step 4) and a scan after 1 Hz pacing (Step 6) to evaluate the dynamism of cell contacts in response to optogenetic stimulation. Traces below the schematic represent recording the vertical displacement of the nanopipette which corresponds to the CM contraction.

The integrated Opto-SICM system is shown in Fig. 1; it combines a hopping-mode SICM with a nanopipette positioned above the sample and an inverted fluorescence microscope with controllable pulsed blue light source for optogenetic triggering. FB-CM co-culture was first observed under a 20x objective with blue light illumination (470 nm, lowest power) to identify the ChR2-positive FBs (ChR2-FB) as seen in Fig. 2, Step1. The nanopipette was carefully positioned above the isolated FB-CM contact region to generate a topography map in hopping mode. For each pair, three topographical images (45 μm x 45 μm, 512 × 512 pixels) were generated prior to the first optical stimulation to evaluate the extent of communication at the contact region. Based on the topographic image, the nanopipette was placed at the highest point of CM. The nanopipette vertical displacement was recorded during CM optical stimulation and in this way the contraction was measured, as the height of the CM changes when it contracts. If the pacing was successful at 0.5 Hz for 60 s, three more topographic images were obtained (Fig. 2, Step 4) and optical stimulation at higher frequency (1 and 1.5 Hz) was performed, followed by three more topographic images (Fig. 2, Step 5/6).

First, we established that direct light stimulation of ChR2-FBs can successfully induce CMs depolarization and contraction. Moreover, the ChR2-FB-CM pairs could be entrained by the light pulses across multiple pacing frequencies (Fig. 2). For a live cell imaging and pacing system, temperature and pH control were confirmed to be important environmental factors influencing the experimental success. Without using a pH balanced buffer or maintaining the proper temperature through the perfusion system with an external heater and on-site thermal probe, the optical pacing success can by 16.6% lower for identical plating conditions (*n* = 23/29, Supplementary Fig. 1a). Upon optical pacing, the detected contraction amplitude of the CMs varied across cell pairs with no specific correlation to the area of contact. However, in all cases, higher pacing rate (1 Hz vs. 0.5 Hz) showed lower contraction amplitude (paired *t*-test, *n* = 11 cell pairs, *p* = 0.0049, Supplementary Fig. 1b).

### Effects of optical pacing on cell-cell contact dynamism in ChR2-myoFB-CM pairs

**Fig. 3:**
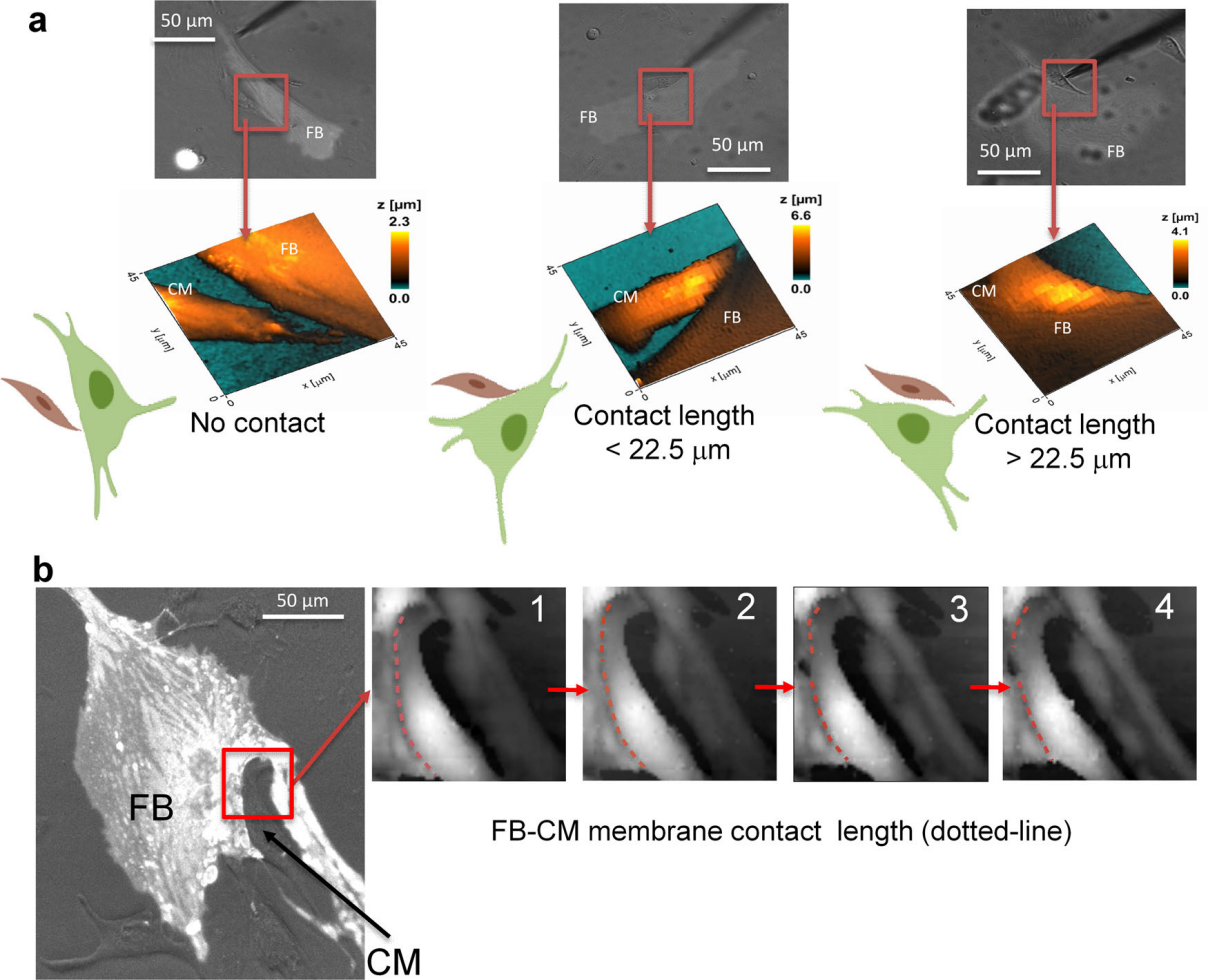
Assessment of cell-cell contact and dynamism of contact for FB-CM pairs. **a** Examples of different status of FB-CM contact reported by the SICM system. All cell pairs appeared to be in-contact through a light microscope, however SICM-based nanotopographic examination revealed various degrees of contact. **b** Contact dynamism resulting from cellular movement during an optogenetic experiment. Images 1 to 4 illustrate the dynamic change in membrane contact between a CM and a FB due to cellular movement. Images were taken every 4 min. Biorender was used for the cell schematics.

**Fig. 4:**
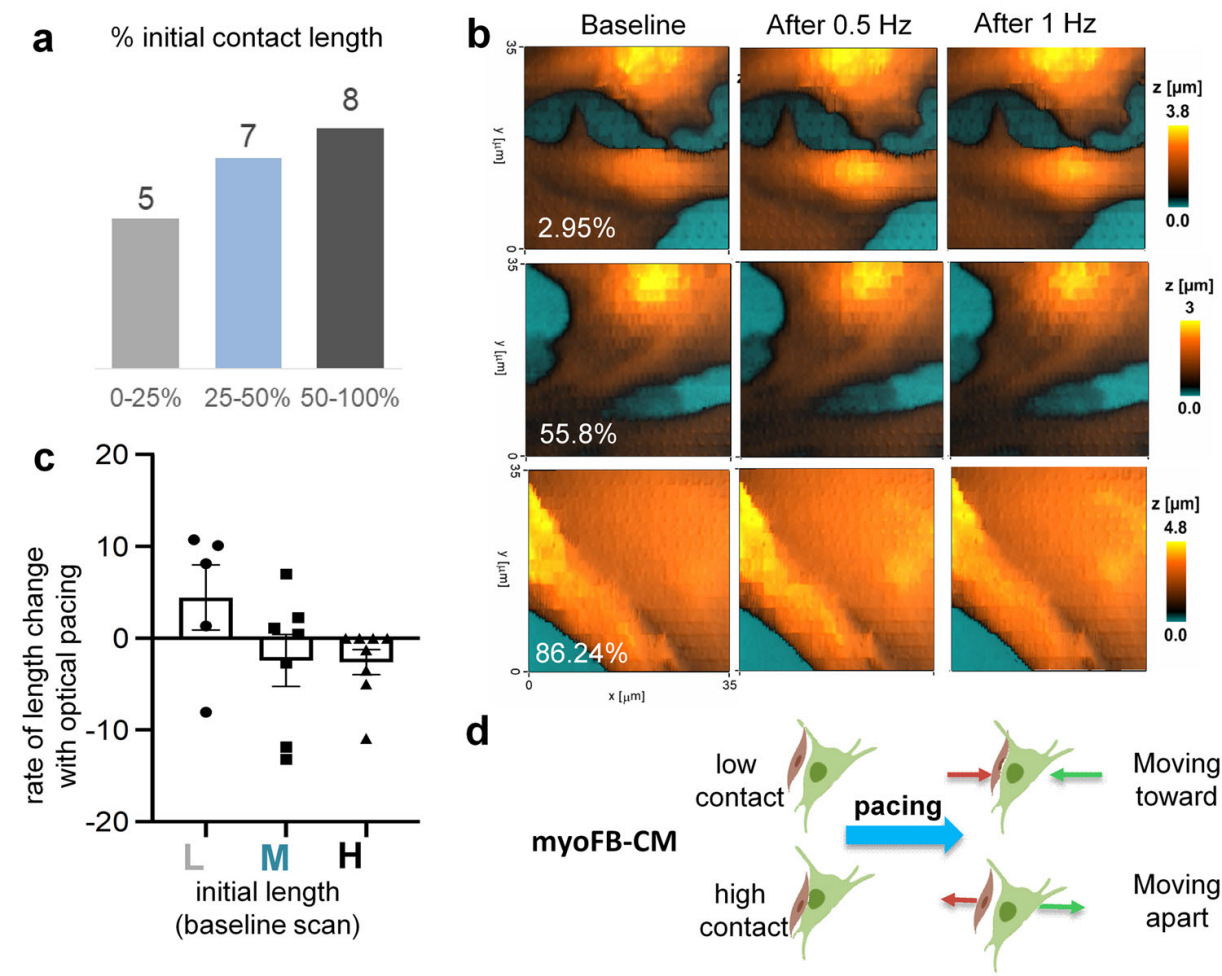
Effects of optical pacing on the contact dynamics in ChR2-myoFB-CM cell pairs (without special treatment, after day 5, FB transitioned to myoFB in these experiments done on glass substrate). **a** Histogram of the percentage of the myoFB-CM contact area in the 20 cell pairs at baseline for low, medium and high area of contact. **b** Select topographic images for the cell pairs at baseline, after 0.5 Hz pacing and after 1 Hz pacing. The indicated are areas of contact at baseline for the three examples shown. **c** Rate of change in contact area with progressive optical pacing (see raw data in Suppl. Fig. 2). Predominantly positive slopes (increase of contact area with pacing frequency) were seen in the low-contact pairs, while the medium and high contact groups saw a slight decrease in contact area with pacing. **d** Schematic illustration of the observed trends in the myoFB-CM cell pairs with pacing, as quantified in panel **c**. Red lines indicate the moving direction of CM, the green lines represent the moving direction of FB. Biorender was used for the cell schematics.

To image cell-cell contacts during optical engagement of heterocellular pairs, bright field images did not provide sufficient resolution and SICM imaging was needed for more detailed view of the contact area. For example, in Fig. 3a, three pairs of FB-CM are shown and all of them have similar contact area in bright field images; however, after scanning with the SICM system, the high-resolution topographic images revealed that the true contact length could vary from zero to longer than 22.5 μm. A series of images acquired from the same heterocellular pairs over time (images were taken every 4 min) using SICM revealed the dynamic nature of cellular contacts (Fig. 3b, Fig. 4).

Over time in culture FBs transdifferentiated into myoFBs, which can be revealed by immunostaining for α-smooth muscle actin; we used myoFB as well in our experiments. Both FBs and myoFBs formed dynamic contacts with CMs. Using custom software CellTrack^55,58^, we tracked the contact movement across the series of recorded SICM images at baseline, after 0.5 Hz, 1 Hz and 1.5 Hz pacing (Fig. 4). The histogram of initial contact length is shown in Fig. 4a for a study of 20 cell pairs subjected to the full optical pacing protocol, binned in terms of the proportion of the contact length to the total membrane length in a frame. Cells were grouped in three groups: low baseline contact length (<25%), medium (25–50%) and high (>50%). Interestingly, with progressive optical pacing, the cell pairs displayed different contact dynamism depending on their pre-stimulation contact area (Fig. 4b–d and Supplementary Fig. 2). Specifically, optical pacing appeared to bring cells closer together (average rate of change in contact area was positive), while higher-contact cell pairs either maintained the area or experienced a small decrease in contact area (Fig. 4c, d).

### Control of FB/myoFB state using SD208

**Fig. 5:**
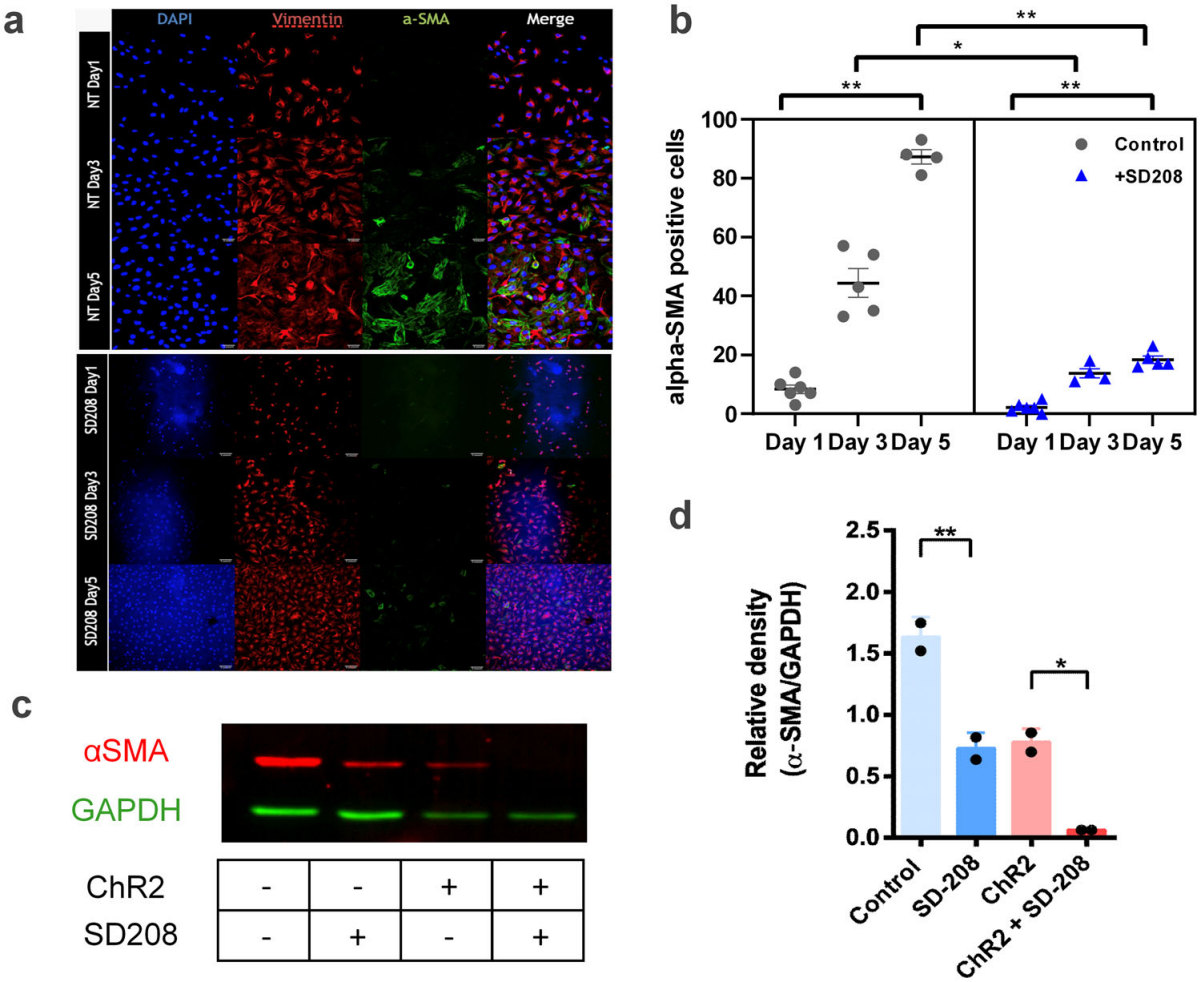
Control of fibroblast phenotype (FB vs. myoFB) with SD208 treatment. **a** Immunocytochemistry images of FB-CM co-cultures with and without SD-208 treatment, labeled for vimentin (red), alpha-SMA (green) and nuclei by DAPI (blue). **b** Quantification of alpha-SMA positive FBs based on fluorescence with time in culture. The SD208 treatment group showed significantly less α-SMA expression compared to untreated, with the difference increasing over time, *p < 0.01; **p < 0.001. **c**, **d** Western blot and quantification of the level of α-SMA protein, normalized by GAPDH. SD208 treatment significantly reduced the level of α-SMA in fibroblasts (switch of myoFB to FB); ChR2-transformation further reduced α-SMA levels, *p < 0.1; **p < 0.05.

In this study of heterocellular coupling via optogenetic probing, we were interested in potential differences between FB-CM and myoFB-CM cell pairs and designed such co-culture experiments (Supplementary Fig. 3). The spontaneous transition of FBs to myoFBs in cell culture was tracked by the expression of α-SMA. Immunocytochemistry revealed that neonatal FBs started to express α-SMA as early as day 3 post-plating and this expression increased with time (Fig. 5a). To create an α-SMA negative FB model, 600 nM SD208, a TGF-β receptor kinase antagonist, was added to the culture media to suppress the conversion of FBs into myoFBs (as seen by the α-SMA synthesis). Indeed, this treatment restricted the fraction of emerging myoFBs to <20% even at day 5, when >80% of the nontreated cultures were myoFBs, Fig. 5b. Western blot results corroborated that SD208 treatment effectively suppressed α-SMA expression in both the native cultures and in ChR2 transfected cultures, (Fig. 5c, d). It is unclear why cells expressing ChR2-eYFP showed a decreased α-SMA expression (Fig. 5d), and it is worth investigating further. In this study, we focused on the effect of SD208 within each group. Interestingly, on day 11, even the SD208 treated group had high α-SMA expression, similar to the no treatment control in Western blot (Supplementary Fig. 4). Therefore, all FB-CM experiments were performed within 5 days in culture.

**Fig. 6:**
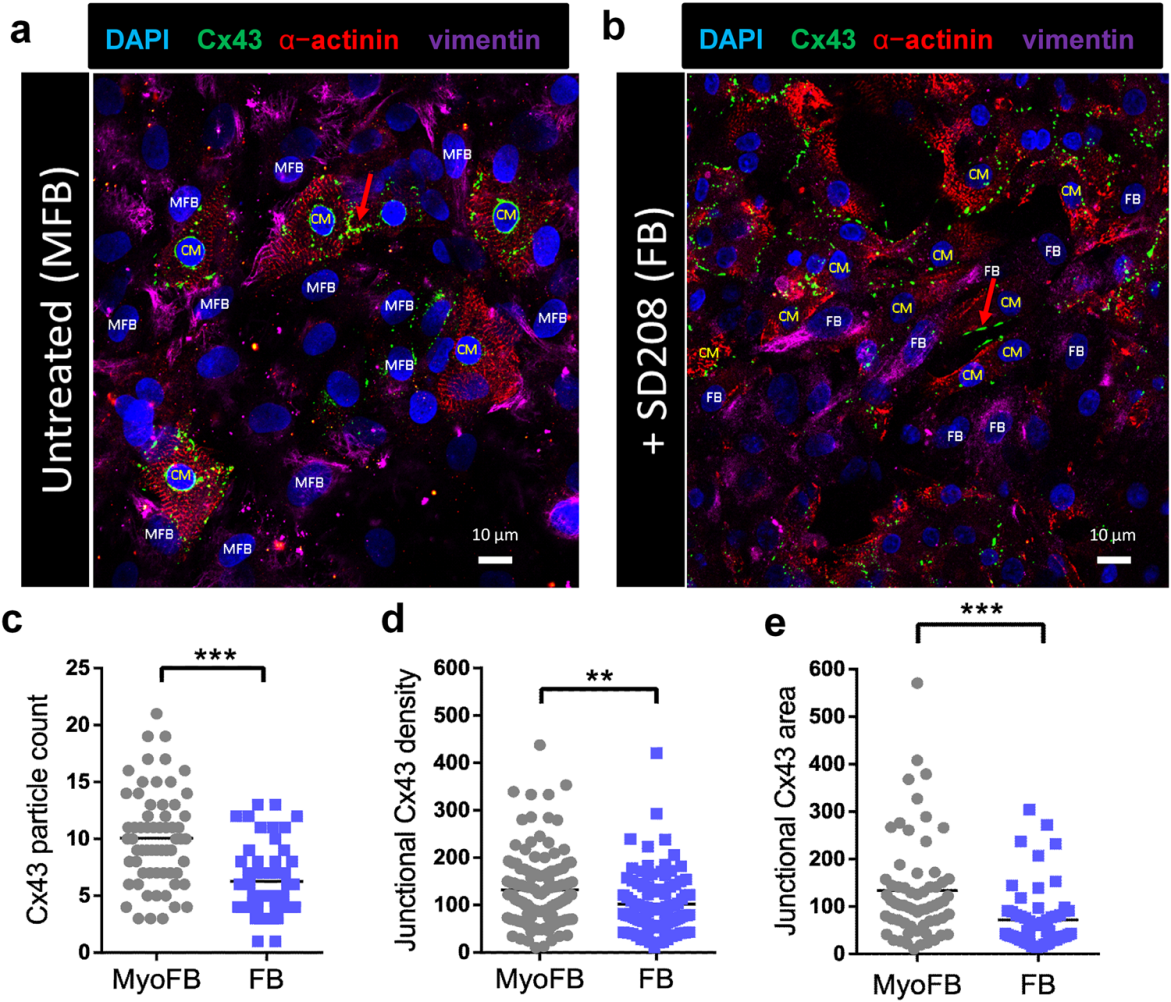
Quantification of gap junction protein Cx43 expression in myoFB-CM and FB-CM pairs by immunocytochemistry. **a**, **b** MyoFB-CM and FB-CM pairs (FB treated with 600 nM SD208 in B) labeled for alpha-actinin (red), Cx43 (green), vimentin (pink) and nuclei by DAPI (blue). Red arrows point to the contact areas, as an example of what has been selected for quantification; **c**–**e** Compared to myoFB, FB showed significantly lower number, lower density and smaller area of C x 43, *p* < 0.01.

As demonstrated by Schultz et al.^55^, myoFB/FB-CM contact dynamism is modulated by the gap junctional protein connexin43 (Cx43). We quantified Cx43 in the FB-CM and myoFB-CM hybrid cell cultures as described in Methods. Vimentin was used to identify the FB/myoFBs and α-actinin to identify the CMs (Fig. 6a, b). In the untreated condition, myoFB-CM cell-cell contact areas were analyzed for pairs formed by a vimentin-positive cell and an α-actinin-positive cell. In untreated cultures, these predominantly represented myoFB-CM pairs, whereas in SD208-treated cultures the pairs were mostly formed by FB and CMs. In FB-CM pairs (following SD208 treatment), Cx43 at contact areas showed significantly lower particle number, junctional Cx43 density and junctional Cx43 area, compared to the untreated pairs formed predominantly by myoFBs and CMs (Fig. 6c–e), as analyzed by particle tracking using ImageJ. Overall, our immunofluorescence analysis showed that myoFBs have higher gap junctional contact with CMs as compared to FBs-CM pairs, in line with previous studies^44,48,59,60^.

### Effects of cellular factors on pacing success

**Fig. 7:**
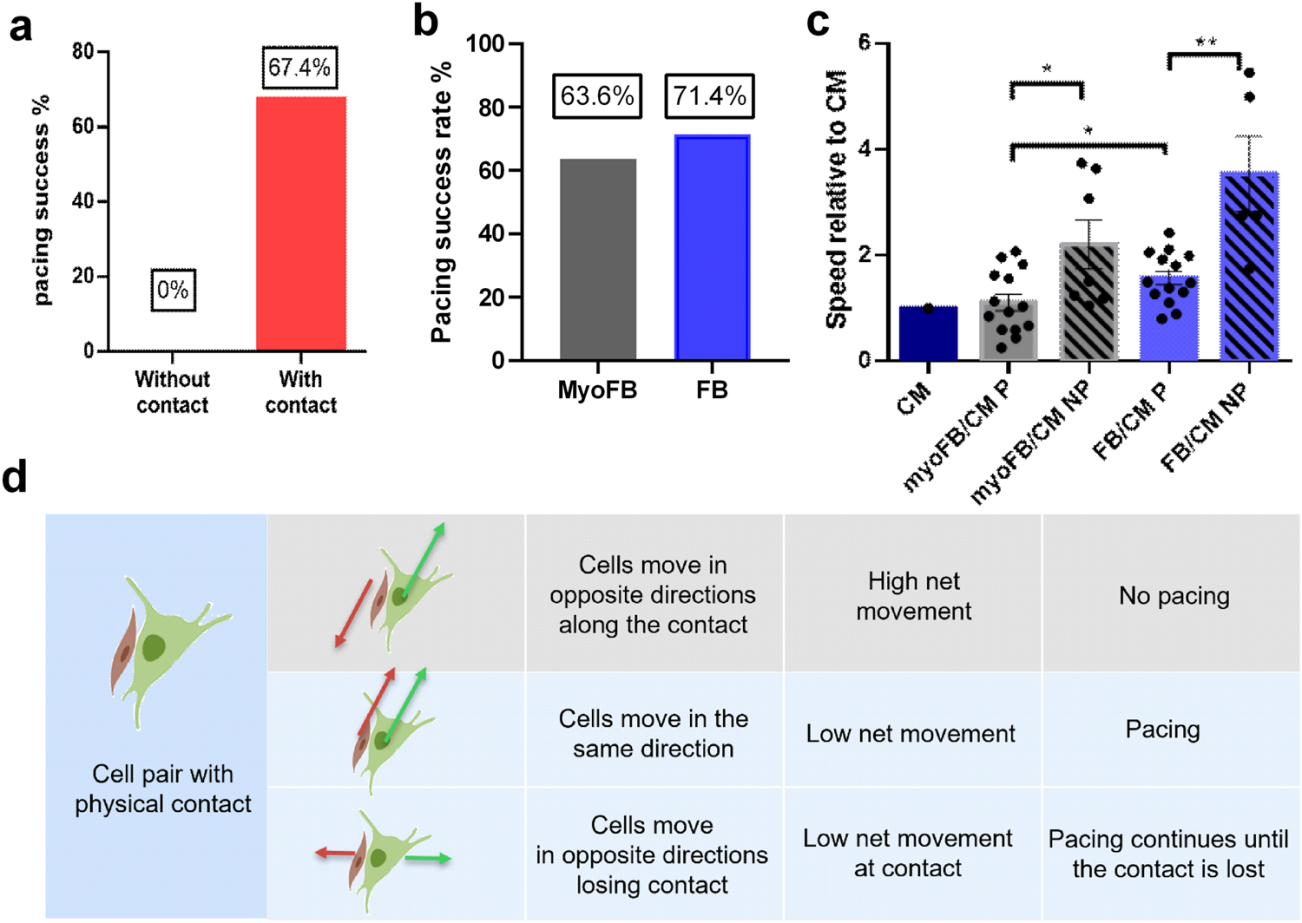
Success of optogenetic pacing in ChR2-myoFB-CM and ChR2-FB-CM cell pairs based on baseline area of contact and dynamics of contact. **a** Physical contact between ChR2-FB/myoFB and CM pairs is necessary for optical pacing. **b** The pacing success rate between myoFB-CM pairs and FB-CM pairs is not significantly different. **c** Relative cell-cell movement is higher in FB-CM pairs compared to myoFB-CM pairs. Cell pairs responsive to optogenetic pacing have lower relative cell-cell movement compared to non-responding cell pairs. *p < 0.05; **p < 0.005. **d** Summary of the effects of contact dynamics on the success of optogenetic pacing for cell pairs with contact. The specific direction of net movement matters; only when low net movement is present, the stable connection can be established. Red arrows indicate the moving direction of CM, the green arrows represent the moving direction of FB. Biorender was used for the cell schematics.

Using the heterocellular models ChR2-FB-CM and ChR2-myoFB-CM, we examined the cellular factors related to contact area and contact dynamism that affect the success of optical pacing, i.e., the transmission of electrical signals from the optogenetically-responsive FBs/myoFBs to the unmodified CMs, Fig. 7. Our initial observation was that CM pacing success was directly related to contact status. Light stimulation failed on all pairs without direct physical contact between the CMs and FBs/myoFBs, as identified using SICM (Fig. 7a). For cell pairs with contact, the overall pacing success rate was 67.4%, with the FB pairs having slightly higher success rate than myoFB pairs (Fig. 7b). As the pacing rate was still less than 100%, other experimental and cellular factors might have influenced the pacing success. We compared the cell contact movement (dynamism) for all studied cell pairs: CM-CM, ChR2-FB-CM and ChR2-myoFB-CM, segregating them based on their responsiveness to optical pacing (P vs. NP). The optogenetically-modified heterocellular cell pairs had higher contact dynamism compared to CM-CM pairs; also, the FB-CM pairs exhibited faster relative movement compared to the myoFB-CM pairs (Fig. 7c). In both heterocellular conditions FB-CM and myoFB-CM, the cell pairs that were responsive to optical pacing had more stable contacts (less movement of the contact) compared to the higher contact dynamics in non-pacing pairs (Fig. 7c, d). In fact, the heterocellular cell pairs that were responsive to optical pacing had similarly stable contacts to homocellular CM-CM pairs. A summary illustration of the contact dynamics conditions and the success of transmitting electrical signals from the FBs/myoFBs to the CMs is shown in Fig. 7d. Maintenance of sufficient physical contact and low net movement were found to be critical for responsiveness to optical stimulation via the non-myocytes.

## Discussion

There is clinical interest in assessing pathological electrical coupling between CMs and non-excitable cells in the heart, myoFBs, which often precipitates cardiac arrhythmias. Furthermore, in cell therapy for cardiac regeneration, proper coupling of the delivered cells to the native tissue is a prime predictor of safety and success of the therapy. Therefore, electrical coupling between cardiomyocytes and heterocellular coupling of cardiomyocytes to other cell types is of great interest. In this study we demonstrate a new direct method (OptoSICM, Fig. 1) for measuring different aspects of functionally coupled CM and FB/myoFB cell pairs. Cell-specific optogenetic triggering via ChR2-FBs and ChR2-myoFBs allowed monitoring of the contractile responses in CMs with simultaneous capture of inter-cellular contact dynamics.

The “gold standard” approach to quantify cell-cell electrical coupling is the dual whole-cell patch clamp^61^. By introducing microelectrodes into adjacent cells, it measures the electrical current across gap junctions. Some limitations of the technique include its restriction to isolated cell pairs with relative high gap junctional resistance and its relatively invasive nature as well as inability to monitor live cell-to-cell contacts. Optogenetic methods have been deployed as contactless cell-specific alternative to assess the functional responses of cardiac tissue via fibroblast actuation using both depolarizing (ChR2) and hyperpolarizing opsins (ArchT or Halorhodopsin)^49,51–54^. For example, OptoGap^62^ was proposed as an all-optical method for assessing coupling in heterocellular cardiac systems based on the light irradiance used to excite. Comprehensive analysis of the effects of optogenetically-induced depolarizing current via ChR2-CFs/myoCFs quantified the functional responses of cardiomyocyte syncytia in terms of spontaneous beating, action potential duration, and conduction velocity using experimental and computational approaches^54^. Using optogenetic tools, it was demonstrated that slowing of cardiac conduction velocity by increased coupling of non-excitable cells to cardiomyocytes is primarily due to capacitive loading^53^. In addition to optogenetic actuators, optogenetic sensors have also been used to probe cardiac heterocellular coupling. VSP2.3 targeted to non-myocytes, revealed transmission of electrical excitation from cardiomyocytes to non-myocytes in the border zone of scar tissue in a Langendorff isolated heart^50^.

While an optogenetic approach provides a valuable cell-specific noncontact tool for interrogating cell-cell coupling, the studies discussed above did not explicitly monitor the physical dynamic changes in the contact zone during pacing and transmission of excitation in these heterocellular systems. The geometry of contact and the dynamic changes in it at the nano- and microscale have dramatic effect on the operation of ion channels and for electro-mechanical coupling in cardiac tissue, as documented by many previous studies^55,63–68^. The nanoscale investigation of the contact zone is done primarily in fixed cells and tissue using electron microscopy or super-resolution optical imaging. SICM represents an exception, allowing for high-resolution view of dynamic changes in the contact area in live cells^55,65,69–72^, (Figs. 2, 3). When combined with optogenetic actuation, OptoSICM provides a fully contact-less high-resolution interrogation technique that is unmatched for real-time tracking of cellular coupling in live cells and tissue. Opto-SICM preserves cell integrity and can study structure-function aspects over time, as demonstrated in this paper. It also has the potential to be incorporated with other techniques such as patch-clamp to non-invasively record cell coupling within a specific region.

Cardiac stress or injury can trigger the production of new FBs from bone-marrow derived cells known as fibrocytes^73^. During the wound healing process, multiple chemokines, cytokines and morphogens such as transforming growth factor beta (TGF-β), together with mechanical stress act upon FBs facilitating their rapid differentiation into myoFBs^42,74^. MyoFBs are the primary phenotype at the site of the injury due to their higher responsiveness and the conditions at the injury site favoring such transformation. Even after healing, a high number of myoFBs can be found in the scar border zone after myocardial infarction. Compared to regular FBs, myoFBs have been shown to exhibit increased levels of gap junctional protein Cx43 and some studies suggest that they may have increased electrical coupling to CMs in the injured heart^44,48,59^, with important impacts on electrotonic loading and arrhythmia occurrence.

In this study, we employed a well-established neonatal FB-CM co-culture, and as shown by others, observed that the rigid substrate in vitro leads to spontaneous transformation of the FBs into myoFBs to 43% at day 3 and to over 85% by day 5 of culture. To control/limit this transition and create FB-CM pairs to compare to the myoFB-CM pairs, we targeted TGF-β signaling. Inhibition of TGF-β has been shown to suppress the synthesis of α-SMA, in TGF-β deficient embryonic FBs^75^. We selected SD208, a highly specific TGF-β receptor I kinase inhibitor, to create a control FB model^23^. By adding SD208, after 96 h incubation, there were 60% less α-SMA positive FBs (Fig. 5). We also confirmed that myoFBs had higher Cx43 expression compared to FBs (Fig. 6).

We leveraged the ability of the Opto-SICM system to generate high resolution images of FB/myoFB-CM contact areas and obtained multiple images from each heterocellular pair over 45 min under varying conditions of optogenetic pacing (Figs. 2, 3). In co-cultures, both CMs and FBs were not stationary, as shown in our previous study^55^. In this study, we found that regardless of FB/myoFB phenotype, on average, the heterocellular pairs that were responsive to optogenetic pacing had significantly more stable contacts and approximately two-fold slower movement compared to non-pacing pairs (Fig. 7). In all studied cases, non-zero area of contact was required for transmission of excitation via optical pacing; as low as 3% contact along the border was seen to yield responsiveness to pacing. We did not observe a significant difference in the ability to pace between FBs and myoFBs as partners to CMs. This is in contrast to expected easier transmission of excitation between myoFB to CMs compared to FB-CM pairs based on Cx43 levels (Fig. 6) and their higher stability of contact area (Fig. 7). Multiple factors can affect actual electrical coupling in addition to the factors listed here; for example, it is difficult to obtain exactly the same population of contact areas for heterocellular pairs involving FBs and myoFBs. Differences in pacing success can be influenced by the specific population of heterocellular pairs studied. Future studies can be designed to control the extent of contact.

In our experiments, FBs and CM were plated sparsely, leaving free space for them to migrate. Speed is a vector variable; for each cell, the net movement is not only determined by absolute distance of movement, but also by the direction of movement. Different movement patterns affect the ability to pace, Fig. 7. Only for low net movement between the two cells, a stable connection was established and pacing - maintained. In future studies, plating FBs and CMs on patterned surfaces could be employed to restrict and/or modulate their net movement and pacing success.

The initial area of contact in the studied heterocellular pairs did not affect the contraction amplitude of the CMs during pacing. However, in all heterocellular pairs higher optical pacing frequencies yielded lower contraction amplitudes in the CMs, consistent with negative frequency-contraction relationship for these immature rat cells, as seen also with electrical pacing^76^. Interesting dynamics were observed at the contact zone after optical pacing at progressively higher frequency, depending on the extent of the initial contact, Fig. 4. Low-contact samples were brought closer together by the progressive electrical engagement via optical pacing. Higher-contact samples experienced net cell movement that either preserved or reduced the area of contact during higher frequency pacing. These complex responses may influence the remodeling of excitation pathways during persistent arrhythmias in the heart, such as tachycardias.

The success rate of pacing in the examined heterocellular pairs likely could be improved further. In some cases, this could be due to immaturity of the neonatal rat cardiomyocytes used in this study. We examined some of the non-pacing pairs with electrical stimulation, during which they also failed to respond. This supported the validity of optogenetic interrogation. Our Western blot results showed possible upregulation of Cx43 expression in both FB and myoFBs upon ChRh2 transduction, as also seen in HEK293 cells and in human induced pluripotent stem-cell-derived cardiomyocytes^29,77^. This is an interesting observation deserving further investigation. Increased electrical coupling between FBs/myoFBs and CMs can influence pacing incidence in both cell types. While it will be important to conduct similar studies using adult cardiomyocytes, it has remained challenging to maintain viable adult CMs in cell culture and have them make connections to FBs. Nevertheless, with optimized cell plating conditions, Opto-SICM could be successfully employed to examine structure-function relationship of both control adult FB-CM and adult myoFB-CM pairs from diseased hearts.

Opto-SICM can be expanded by adding capabilities for all-optical electrophysiology^78^, for comprehensive analysis of voltage and calcium responses. This technique is also valuable for application in cultures consisting of human induced pluripotent stem cells, alongside ChR2-transduced FB, or adding cardiomyocytes from diseased hearts, after myocardial infarction or with known genetic mutations that may affect coupling. Additionally, Opto-SICM can potentially be used for studying other excitable cells, such as neurons and smooth muscle cells, alongside either homo-cellular or hetero-cellular cell coupling, as other optogenetics-based studies have suggested^32–34^.

In conclusion, we have successfully established neonatal rat FB/CM and myoFB/CM models and a standard workflow for the Opto-SICM system. Our data demonstrate that Opto-SICM can generate real-time high-resolution images of interacting living cells while optogenetic actuation is applied in a cell-specific manner. Therefore, the system is ideal for studying cell to cell contact dynamics and how such dynamism influences the transmission of electrical signals between cell types.

## Methods

### Neonatal rat fibroblast and cardiomyocytes isolation

Neonatal rat ventricular CMs and FBs were isolated from 1 or 2-day old Sprague-Dawley rat pups using the MACS neonatal heart dissociation kit as previously described^79^. The procedure was in accordance with the guidelines of the Home Office Animal (Scientific Procedures) Act from 1986 of the United Kingdom. CMs and FBs were isolated and cultured separately for 48 h in M199 media with 10% Newborn calf serum 1% L-Glutamax (100x), 0.5% Vitamin B12 stock (0.4 mg/ml) and 0.5% Antibiotic/antimycotic solution. Monocultures of CMs were seeded at a density of 50 000 cells onto 35 mm MatTek dishes (MatTek, Ashland, MA, USA). Some FBs were cultured in the medium containing 600 nM SD208 (TOCRIS, UK) to prevent myofibroblast transition. After 48 h, myofibroblasts and SD208 treated fibroblasts were dissociated from the flasks with trypsin-EDTA and added to sparse CM cultures for further 48 h prior to being used in Opto-SICM measurements. FBs and CMs were kept in SD208-containing medium during co-culturing.

### Channelrodopsin2-YFP fibroblast transformation

Neonatal rat FBs were optogenetically transformed right after isolation using custom-made Adenovirus-ChR2(H134R)-eYFP (developed based on Addgene plasmid 20940, courtesy of K. Deisseroth^80^) at MOI 250 in OPTI-MEM Reduced Serum Medium (Gibco, USA) using infection in suspension for two hours, as described previously^51,81^. Transformed fibroblasts were re-suspend and plated in M199 media with 10% Newborn Calf Serum (Sigma, UK), 1% L- Glutamax (100x), 0.5% Vitamin B12 stock (0.4 mg/ml) and 0.5% Antibiotic/ antimycotic solution +/– 600 nM SD208 for two days before co-culturing.

### Immunocytochemistry and confocal microscopy

Immunofluorescence microscopy of alpha smooth muscle actin, αSMA, (anti- mouse MA511547, 1:500; Thermo Fisher Scientific, USA), Cx43 (anti-rabbit C6219, 1:1000; MilliporeSigma, USA), and DAPI (33342, 1:1000; Thermo Fisher Scientific) were used to assess myofibroblast transformation during culturing and to examine Cx43 expression at hetero-cellular cell contacts.

### Protein extraction and Western blotting

Fibroblasts were plated in 35-mm dishes (0.5 million cells per dish) and cultured for 96 h with or without SD208 treatment. Cells were subsequently washed with cold PBS and the protein was extracted using RIPA lysis buffer containing protease inhibitors. Collected lysates were sonicated for 2 min at 0 °C and centrifuged for 10 mins at 14,800 rpm. Protein concentrations were determined using a BCA Protein Assay according to the manufacturer’s protocol (Pierce, Rockford, IL, USA). Proteins were loaded into 10% gel and were then transferred to PVDF membranes (Bio-Rad, USA) using the Trans-Blot Turbo System (Bio-Rad, USA). Post transfer, membranes were blocked with 5% skimmed milk (MilliporeSigma, USA) in TBS-T (20 mM Tris, 150 mM NaCl, containing 0.05% Tween-20, pH = 7.4), for one hour and incubated with primary antibody–α-SMA (1:1000, mouse monoclonal, Dako, Denmark) and GAPDH (1:200, polyclonal antibody, Invitrogen, USA), overnight. On day two, after washing with TBS-T the membrane was incubated with the secondary antibody (Donkey anti-rabbit-HRP, Cell Signaling Technology, USA; donkey anti-mouse HRP, Cell Signaling Technology, USA or AlexaFluor donkey anti-mouse 488, A21202, Invitrogen and AlexaFluor donkey anti-rabbit 546, A10040, Invitrogen) diluted 1:1000 in 5% skimmed milk in TBS-T for 1 hour at RT. Membranes were washed again and exposed to clarity enhanced chemiluminescence (ECL) reagent (Bio-Rad, USA) for 5 mins at RT and visualized. Fluorescent blots were imaged using a Bio-Rad ChemiDoc MP at the appropriate wavelength. Quantification of protein band intensities was conducted using ImageJ. The α-SMA protein bands were normalized to GAPDH bands.

### Integrated Opto-SICM system

As shown in Fig. 1, the integrated Opto-SICM system combines a hopping-mode SICM with a nanopipette from the top and an inverted fluorescence microscope with controllable pulsed blue light source for optogenetic triggering.

The dish containing a FB-CM co-culture was mounted on the microscope stage and the cells were first observed under a 20X objective with blue light (470 nm, lowest power) illumination to identify the ChR2-positive FBs (ChR2-FB) as seen in Fig. 2, Step1. Only pairs of a ChR2-FB and a CM not connected to other cells were selected to study. Preliminary testing with pulsed blue light was done prior to scanning the contact area between the two cells. The nanopipette (~100 nm inner diameter, ID, 100 MΩ resistance) was filled with Tyrode solution containing NaCl 135 mM, MgCl_2_1 mM, glucose 5 mM, HEPES 5 mM, KCL 5.4 mM, CaCl_2_ 1.5 mM, NaH_2_PO_4_ 0.33 mM; pH 7.4, all agents by Sigma, USA. The nanopipette was carefully positioned above the cell pair contact region to generate a topography map in hopping mode^7^ (2200 nm vertical displacement). For each pair, three topographical images (45 μm × 45 μm, 512 × 512 pixels) were generated prior to the first optical stimulation to evaluate the extent of communication at the contact region. Following scanning, the nanopipette was re-positioned above the highest point of CM according to the topographic image. Upon CM contraction, the nanopipette resistance at the tip would rise, initiating displacement of the pipette, which was used for recording of cardiomyocyte contraction. Optical stimulation was achieved using 10 ms pulses of blue light (470 nm), at approximately 0.2 mW/mm^2^, delivered through the objective of the epifluorescence set-up (Fig. 2 Step3). First optical pacing of the ChR2-FB-CM pair was applied at 0.5 Hz for 60 sec, after which three more topographic images were obtained (Fig. 2, Step 4). If selected CM contracted in response to light-activated ChR2-FB at 0.5 Hz, then additional higher frequency light stimulation (1 Hz, 60 s, Fig. 2, Step 5) with the same light conditions was performed, followed by three topographic image scan (Fig. 2, Step 6). For some cell pairs this procedure was repeated also for 1.5 Hz pacing.

### Tracking changes in contact area

Depending on the contractile status of cardiomyocytes, a series of six (for non-pacing) or nine (for pacing) images per cell pair were imported into a custom-made cellular movement quantification software, CellTrack^58^. Upon manual selection of the contact region, the software automatically labels intracellular features and tracks those features across the series of images. The movement data were converted into µm/min based on image scales (45 µm x 45 µm, 512 × 512 pixels).

### Quantitative analysis of Cx43 at hetero-cellular junctions

Following immunocytochemical labelling of Cx43, particle analysis was carried out using Particle Analysis plugin in Fiji (ImageJ). Hetero-cellular pairs were first identified as a neonatal cardiomyocyte (CM) being attached to either a myoFB or a FB. myoFBs were selectively identified as both vimentin and α-SMA-positive cells in control, untreated condition. Conversely, in SD208 treated cultures, selective identification of FBs was achieved by targeting vimentin-only positive FB. Images were initially converted to grayscale and then a threshold was applied to binarize the images (black = 0, white = 255 gray scale level). A manual drawing tool was used to outline a region of interest (ROI) of CM-FB junctional area with Cx43 immunofluorescence particles. In this junctional section, the following parameters were measured: Cx43 particle count, Cx43 integrated density, average Cx43 particle size and% area immune-positive for Cx43.

### Statistics and reproducibility

All experiment data was plotted and analyzed in GraphPad Prism 9 software (GraphPad Software Inc., USA). Data was analyzed for normality (Shapiro–Wilk normality test) and statistical significance (t-test: unpaired, parametric) and was presented as mean ± standard error of the mean. Statistically significant difference was expressed as: *p < 0.05; **p < 0.01; ***p < 0.001.

### Reporting summary

Further information on research design is available in the Nature Portfolio Reporting Summary linked to this article.

### Supplementary information

Supplementary Information

Description of Additional Supplementary Data

Supplementary Data

reporting summary

## Data Availability

The source data for graphs and charts in available as Supplementary Data and any remaining information can be obtained from the corresponding author upon reasonable request.
