# Supplementary material for: Opto-SICM framework combines optogenetics with scanning ion conductance microscopy for probing cell-to-cell contacts
Source: Commun Biol. 2023 Nov 8;6:1131. doi: 10.1038/s42003-023-05509-3 (PMC10632396; doi:10.1038/s42003-023-05509-3)
Supplement: Supplementary file 1 — Supplementary Information [file 42003_2023_5509_MOESM1_ESM.pdf]

SUPPLEMENT

for

Opto-SICM framework combines optogenetics with scanning ion conductance microscopy for probing cell-to-cell contacts.

Qianqian Song[1], Anita Alvarez-Laviada[1], Sarah E. Schrup[2], Benedict Reilly-O'Donnell[1], Emilia Entcheva[2#], Julia Gorelik[1#]

[1] Imperial College London, Du Cane road W12 0NN, London UK;

[2] Department of Biomedical Engineering, George Washington University, Washington, DC, USA.

Running Title: *Opto-SICM framework to study heterocellular connections*

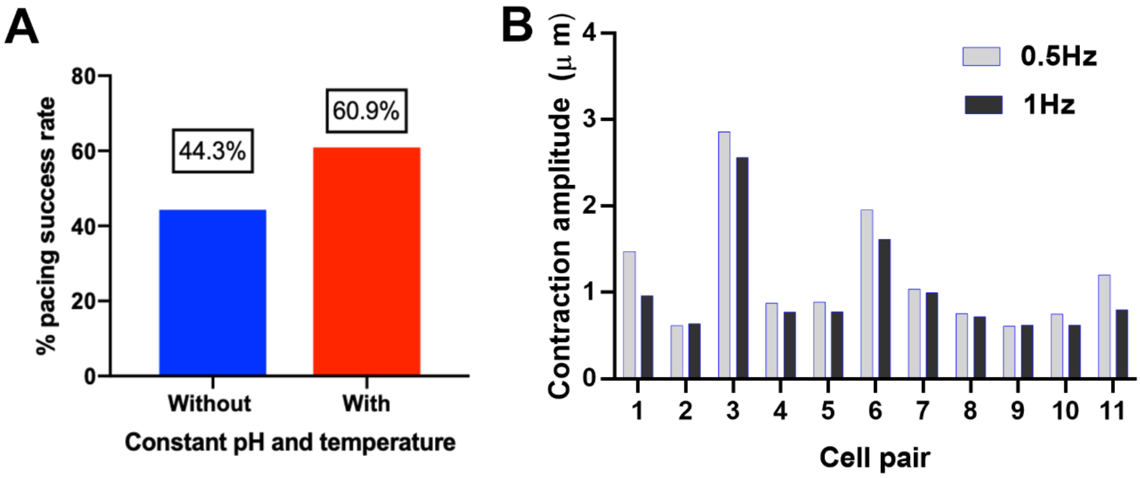

Supplementary Figure 1
Optimizing the Opto-SICM system and effects of pacing on contraction amplitude. A. After implementing constant pH and temperature control, pacing success rate increased by 16.6%. B. Pacing amplitude was significantly lower at 1Hz pacing compared to 0.5Hz pacing (n=11, p=0.0049).

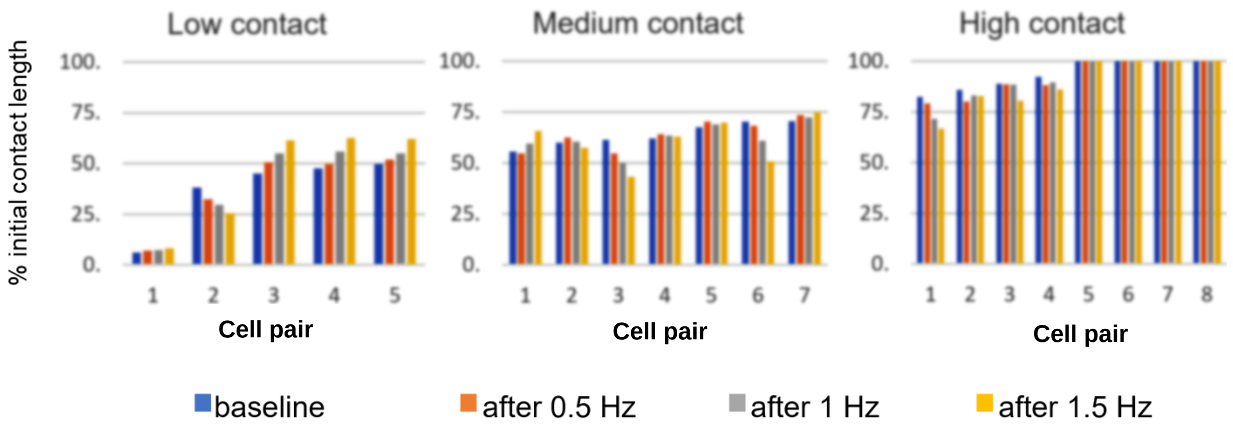

Supplementary Figure 2
Change in % in contact area with optogenetic pacing (n=20) in myoFB-CM cell pairs. Low, medium and high contact are defined as (<25%), (25% to 50%) and (>75%), as in Figure 4.

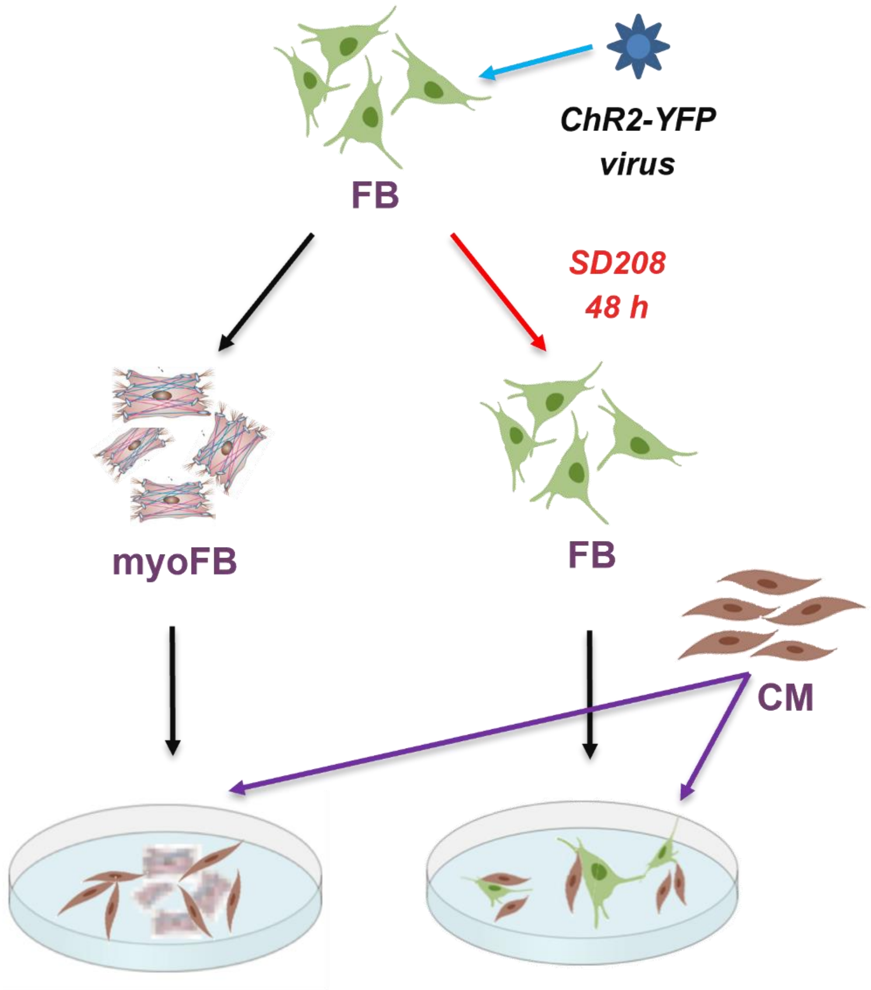

Supplementary Figure 3. Schematic for developing in vitro cardiomyocytes with fibroblast/myofibroblast model. SD208 was effective within 96 hours of incubation, therefore, the experimental incubation period has been limited to 96 hours.

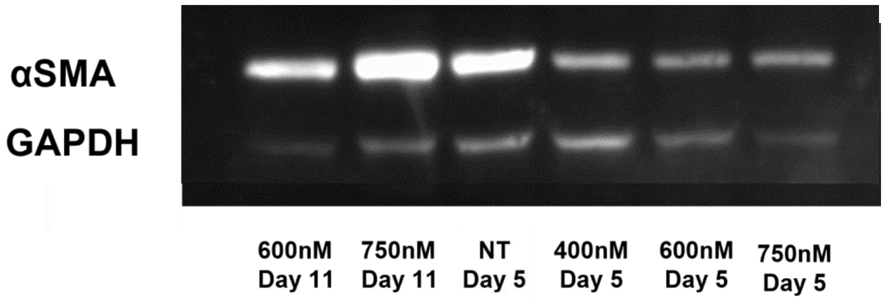

Supplementary Figure 4. Western blot for a-SMA tested on cell treatment with SD208 for 5 days or 11 days. SD208 has significant inhibitory property on cell treated for 5 days in comparison with no treatment (NT) control; effect of the treatment was lost around day 11.
